# Niclosamide enhances abiraterone treatment via inhibition of androgen receptor variants in castration resistant prostate cancer

**DOI:** 10.18632/oncotarget.8493

**Published:** 2016-03-30

**Authors:** Chengfei Liu, Cameron Armstrong, Yezi Zhu, Wei Lou, Allen C. Gao

**Affiliations:** ^1^ Department of Urology, University of California Davis, CA, USA; ^2^ Graduate Program in Pharmacology and Toxicology, University of California Davis, CA, USA; ^3^ UC Davis Comprehensive Cancer Center, University of California Davis, CA, USA

**Keywords:** prostate cancer, niclosamide, abiraterone, androgen receptor variant, resistance

## Abstract

Considerable evidence from both clinical and experimental studies suggests that androgen receptor variants, particularly androgen receptor variant 7 (AR-V7), are critical in the induction of resistance to enzalutamide and abiraterone. In this study, we investigated the role of AR-V7 in the cross-resistance of enzalutamide and abiraterone and examined if inhibition of AR-V7 can improve abiraterone treatment response. We found that enzalutamide-resistant cells are cross-resistant to abiraterone, and that AR-V7 confers resistance to abiraterone. Knock down of AR-V7 by siRNA in abiraterone resistant CWR22Rv1 and C4-2B MDVR cells restored their sensitivity to abiraterone, indicating that AR-V7 is involved in abiraterone resistance. Abiraterone resistant prostate cancer cells generated by chronic treatment with abiraterone showed enhanced AR-V7 protein expression. Niclosamide, an FDA-approved antihelminthic drug that has been previously identified as a potent inhibitor of AR-V7, re-sensitizes resistant cells to abiraterone treatment *in vitro* and *in vivo*. In summary, this preclinical study suggests that overexpression of AR-V7 contributes to resistance to abiraterone, and supports the development of combination of abiraterone with niclosamide as a potential treatment for advanced castration resistant prostate cancer.

## INTRODUCTION

Castration resistant prostate cancer (CRPC) is characterized by increased activation and/or overexpression of androgen receptor (AR) resulting in the transcription of downstream target genes and tumor progression despite castrate levels of androgen in the patient. At this stage of prostate cancer progression, there are currently several agents in use to stop or reverse tumor growth. Amongst these is abiraterone. Abiraterone is a steroidogenesis inhibitor of the androgen synthesis pathway and functions by blocking CYP17A1 activity and preventing the conversion of pregnenolone to dihydrotestosterone (DHT) [1, 2]. This results in significant loss of androgen production in peripheral tissues and loss of production of precursors needed for intratumoral androgen synthesis. Recently, abiraterone was discovered to be converted by 3β-hydroxysteroid dehydrogenase (3β-HSD) to a more active Δ4-abiraterone (D4A) form which blocks multiple steroidogenic enzymes and antagonizes the androgen receptor [3]. Abiraterone treatment in patients who had progressed after docetaxel therapy demonstrated a 3.9 month survival benefit and chemotherapy-naïve patients had a 4.4 month survival benefit with abiraterone [4–6]. It is unfortunate, however, that one-third of all patients display primary resistance to abiraterone treatment, and all patients with initial response progress by 15 months of abiraterone treatment [4].

Although enzalutamide and abiraterone have achieved significant outcomes in the clinic [4, 7], the controversy of sequential treatment for late stage prostate cancer patients remains [8–13]. Moderate effects of sequential treatment of enzalutamide and abiraterone in clinical patients revealed the existence of cross resistance between enzalutamide and abiraterone. This brings to light an urgent need to identify the cross resistance mechanisms involved in order to discover biomarkers and treatment strategies that can overcome the resistance.

Several pathways have been identified which may play a role in the development of abiraterone resistance, many of which involve re-activation of androgen synthesis or changes in androgen receptor signaling [14–18]. Of interest in the presented study, is the role of androgen receptor variants. These variants are produced due to alternative splicing or by genome rearrangement and oftentimes result in loss of the AR ligand binding domain leading to constitutive activation of the receptor [19–22]. Of these variants, androgen receptor variant 7 (AR-V7) is the most widely studied and its expression is associated with resistance to multiple therapeutics including enzalutamide and abiraterone[14, 16, 23–25]. Clinically, Antonarakis et al. demonstrated that patients treated with enzalutamide or abiraterone that had AR-V7 expression displayed significantly lower PSA response, shorter progression-free and overall survival compared to men without AR-V7 [26].

Niclosamide, an FDA-approved antihelminthic, has been identified as a potent AR-V7 inhibitor in prostate cancer cells [27]. Niclosamide promotes AR-V7 protein degradation, possibly through activation of the ubiquitin proteasome pathway. Furthermore, it reduces AR-V7 transcriptional activity and reverses enzalutamide resistance [27, 28]. However, the experimental evidence of the roles of AR-V7 in the responsiveness to abiraterone treatment and the effects of niclosamide on abiraterone treatment remain unknown. In the present study, we found that enzalutamide-resistant cells are cross-resistant to abiraterone and that AR-V7 promotes abiraterone resistance. Abiraterone resistant prostate cancer cells generated by chronical treatment with abiraterone showed significantly enhanced AR-V7 protein expression. Furthermore, niclosamide treatment sensitized abiraterone-resistant cells to abiraterone through AR-V7 inhibition. Of particular importance was the finding that niclosamide was capable of reversing abiraterone resistance *in vivo* through the oral administration. This study provides insight into prostate cancer cross-resistance and demonstrates a novel therapy for overcoming abiraterone resistance by niclosamide. Data contained herein are a critical step towards the development of treatment strategies for patients with advanced castration resistance prostate cancer.

## RESULTS

### C4-2B MDVR cells are cross resistant to abiraterone

**Figure 1:**
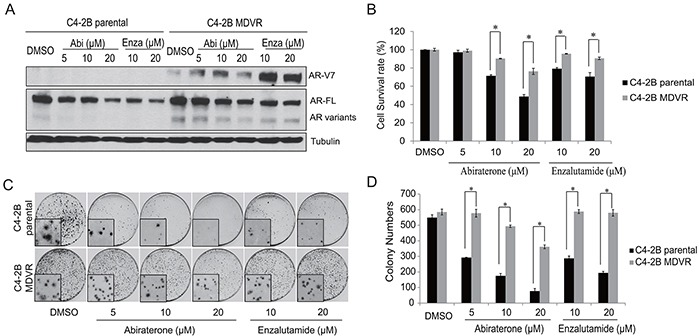
Enzalutamide resistant prostate cancer cells are cross resistant to abiraerone **A.** C4-2B parental and C4-2B MDVR cells were treated with different dose of abiraterone (5μM, 10 μM and 20 μM) or enzalutamide (10 μM and 20 μM) and total cell lysates were harvested and subjected to western blot. **B.** C4-2B parental and C4-2B MDVR cells were treated with different doses of abiraterone (5μM, 10 μM and 20 μM) or enzalutamide (10 μM and 20 μM) for 48 hours and total cell numbers were counted and cell survival rate was calculated. **C.** Colonogenic assay was performed. Pictures were taken under microscope (inside panel). **D.** The colonies were counted and results are presented as means ± SD of 2 experiments performed in duplicate. Results for other panels are presented as means ± SD of 3 experiments performed in duplicate. *P<0.05

In present study, we generated enzalutamide resistant C4-2B MDVR cells [29]. C4-2B MDVR cells have high expression of AR variants, including AR-V7, compared to parental C4-2B cells and expression of these variants cannot be inhibited by either abiraterone or enzalutamide (Figure 1A). This suggests AR-V7 might be the underlying mechanism of cross resistance to both abiraterone and enzalutamide. To test this hypothesis, we investigated the differences in response to abiraterone between C4-2B parental and C4-2B MDVR cells. Both cell lines were treated with varying concentrations of abiraterone or enzalutamide for 48 hours and cell numbers were determined. As shown in Figure 1B, C4-2B parental cells are sensitive to both abiraterone and enzalutamide, while C4-2B MDVR cells showed a reduced response to abiraterone and resistance to enzalutamide. These results were also confirmed by clonogenic assay. As depicted in Figure 1C and 1D, abiraterone and enzalutamide significantly inhibited C4-2B parental cell colony formation ability while both drugs had limited effects on C4-2B MDVR cells. Collectively, the above results suggest a cross resistance phenomenon is present between enzalutamide and abiraterone.

### AR-V7 confers resistance to abiraterone in prostate cancer

**Figure 2:**
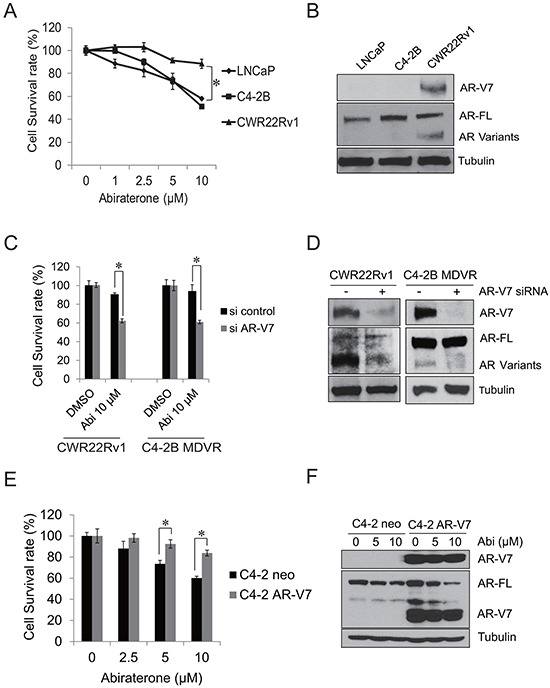
AR-V7 confers abiraterone resistance in prostate cancer **A.** CWR22Rv1, C4-2B and LNCaP cells were treated with different concentrations of abiraterone for 48 hours, total cell numbers were counted and cell survival rate was calculated. **B.** whole cell lysates from LNCaP, C4-2B and CWR22Rv1cells were extracted, AR-V7 and full length AR were examined by western blot. **C–D.** CWR22Rv1 or C4-2B MDVR cells were transiently transfected with AR-V7 siRNA and then treated with 10 μM abiraterone for 3 days, total cell numbers were counted and cell survival rate (%) was calculated and the AR-V7 knock down efficiency was examined by western blot. **E.** C4-2 neo or C4-2 AR-V7 cells were treated with different concentrations of abiraterone for 48 hours, total cell numbers were counted and cell survival rate (%) was calculated. **F.** C4-2 neo or C4-2 AR-V7 cells were treated with different concentration of abiraterone for 48 hours, whole cell lysates were subjected to western blot. Results are presented as means ± SD of 3 experiments performed in duplicate. *P<0.05 Abi: Abiraterone.

To further examine the sensitivity of prostate cancer cells to abiraterone, LNCaP, C4-2B and CWR22Rv1 cells were treated with different concentrations of abiraterone for 48 hours. CWR22Rv1 cells are more resistant to abiraterone treatment than LNCaP and C4-2B cells (Figure 2A). The AR-V7 expression was examined by western blot. As shown in Figure 2B, CWR22Rv1 cells express significantly higher AR-V7 than LNCaP and C4-2B cells. Previous studies have shown that AR-V7 might be an important player for driving enzalutamide resistance in prostate cancer cells [24, 27]. To examine whether it also induces abiraterone resistance, CWR22Rv1 and C4-2B MDVR cells were transiently transfected with AR-V7 siRNA and subsequently treated with 10 μM abiraterone for 3 days. As shown in Figure 2C, knockdown of AR-V7 significantly enhanced abiraterone treatment in both CWR22Rv1 and C4-2B MDVR cells. The effects of AR-V7 knockdown were examined by western blot (Figure 2D). To determine whether overexpression of AR-V7 confers resistance to abiraterone, we generated AR-V7 overexpressing C4-2 AR-V7 cells by stably expressing AR-V7 in C4-2 cells. C4-2 neo and C4-2 AR-V7 were treated with different concentrations of abiraterone for 48 hours and cell numbers were determined. C4-2 AR-V7 cells exhibited resistance to abiraterone treatment compared to C4-2 neo cells (Figure 2E). Abiraterone inhibits full length AR but not AR-V7 expression in C4-2 AR-V7 cells (Figure 2F). These results demonstrate that overexpression of AR-V7 confers resistance to abiraterone in prostate cancer cells.

### Niclosamide inhibits AR-V7 expression and enhances abiraterone treatment *in vitro*

**Figure 3:**
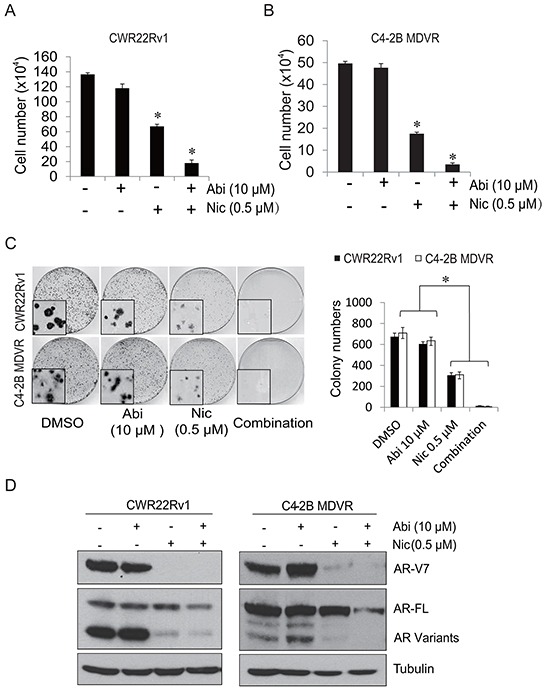
Niclosamide enhanced abiraterone treatment *in vitro* **A–B.** CWR22Rv1 cells or C4-2B MDVR cells were treated with 0.5 μM niclosamide with or without 10 μM abiraterone in media containing FBS and cell numbers were counted after 48 hours. Results are presented as means ± SD of 3 experiments performed in duplicate. **C.** Clonogenic assays were performed. Colonies numbers were counted and results are presented as means ± SD of 2 experiments performed in duplicate. **D.** CWR22Rv1 and C4-2B MDVR cells were treated with DMSO, 10 μM abiraterone, 0.5 μM niclosamide or combination for 48 hours, total cell lysates were collected and subjected to western blot. *P<0.05 Abi: Abiraterone. Nic: Niclosamide.

Having demonstrated that AR-V7 also confers resistance to abiraterone treatment, we next determined whether niclosamide improves abiraterone treatment by inhibition of AR-V7 expression in prostate cancer cells. To examine if niclosamide enhances abiraterone treatment *in vitro*, CWR22Rv1 or C4-2B MDVR cells were treated with 0.5 μM niclosamide with or without 10 μM abiraterone for 2 days and total cell numbers were counted. As shown in Figure 3A and 3B, 10 μM abiraterone had limited effects on cell growth, 0.5 μM niclosamide inhibited cell growth, and the combination treatment significantly inhibited cell growth more than either treatment on its own. The results were confirmed by clonogenic assay: combination of niclosamide with abiraterone significantly inhibited colony number and reduced colony size in CWR22Rv1 and C4-2B MDVR cells (Figure 3C). Western blots were performed to determine if combination treatment with niclosamide and abiraterone inhibit AR-V7 expression. As shown in Figure 3D, 0.5 μM niclosamide inhibited AR-V7 protein expression but had only moderate effects on full length AR. Combined niclosamide and abiraterone treatment not only further inhibited AR variants expression, but also inhibited full length AR expression in both CWR22Rv1 and C4-2B MDVR cells. Taken together, these results suggest niclosamide can enhance abiraterone treatment by inhibition of AR-V7 expression *in vitro*.

### C4-2B abiraterone resistant cells generated by chronic treatment with abiraterone showed enhanced expression of AR-V7

**Figure 4:**
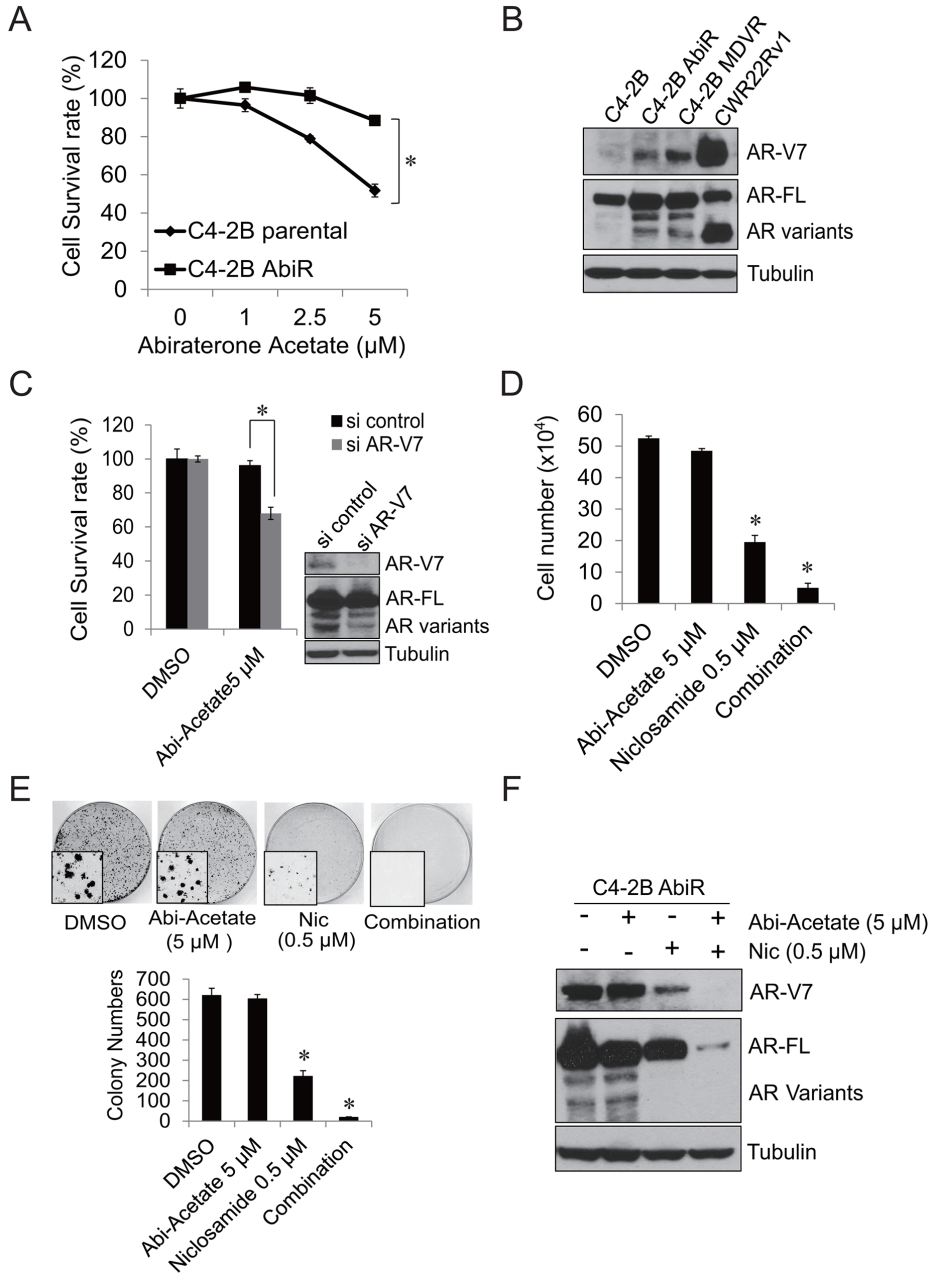
C4-2B cells chronically treated with abiraterone acetate express AR-V7 **A.** C4-2B parental and C4-2B AbiR cells were treated with different concentrations abiraterone acetate for 3 days, total cell number was counted and cell survival rate was calculated. **B.** C4-2B parental, C4-2B AbiR, C4-2B MDVR and CWR22Rv1 cells were cultured in media containing FBS for 3 days, total cell lysates were collected and subjected to western blot. **C.** C4-2B were transiently transfected with AR-V7 siRNA and then treated with 5 μM abiraterone acetate for 3 days. Total cell numbers were counted and cell survival rate (%) was calculated. The AR-V7 knock down efficiency was examined by western blot. **D.** C4-2B AbiR cells were treated with 0.5 μM niclosamide with or without 5 μM abiraterone acetate in media containing FBS and cell numbers were counted after 48 hours. Results are presented as means ± SD of 3 experiments performed in duplicate. **E.** C4-2B AbiR cells were treated with 0.5 μM niclosamide with or without 5 μM abiraterone acetate in media containing FBS and clonogenic assays were performed, colony pictures were taken under microscope. **F.** C4-2B AbiR cells were treated with DMSO, 5 μM abiraterone acetate, 0.5 μM niclosamide or combination for 48 hours. Total cell lysates were collected and subjected to western blot. *P<0.05 Abi-acetate: Abiraterone acetate. Nic: Niclosamide.

To further examine that AR-V7 confers abiraterone resistance, we generated an abiraterone resistant prostate cancer cell line by continuous culture of C4-2B cells in media containing abiraterone acetate. As shown in Figure 4A, after 12 months of being cultured in abiraterone acetate containing medium, C4-2B AbiR (C4-2B abiraterone resistant) cells developed resistance to abiraterone acetate. Abiraterone acetate at 5 μM significantly suppressed the growth of C4-2B parental cells, but had limited effects on C4-2B AbiR cells. We then examined the AR-V7 levels in C4-2B AbiR cells. As shown in Figure 4B, C4-2B AbiR cells express significantly higher levels of AR-V7 protein compared to C4-2B parental cells. Knock down of AR-V7 expression significantly re-sensitized C4-2B AbiR cells to abiraterone acetate (Figure 4C). To find out if niclosamide could enhance abiraterone treatment in C4-2B AbiR cells, C4-2B AbiR cells were treated with DMSO, 5 μM abiraterone acetate, 0.5 μM niclosamide or their combination for 48 hours. Niclosamide significantly suppressed cell growth in C4-2B AbiR cells, while combination treatment further enhanced the effects (Figure 4D). The results were also confirmed by clonogenic assay (Figure 4E). Niclosamide significantly inhibited AR-V7 expression, combination of abiraterone and niclosamide further suppressed both full-length AR and AR-V7 expression in C4-2B AbiR cells (Figure 4F).

### Niclosamide synergizes abiraterone treatment *in vivo* through the oral administration

**Figure 5:**
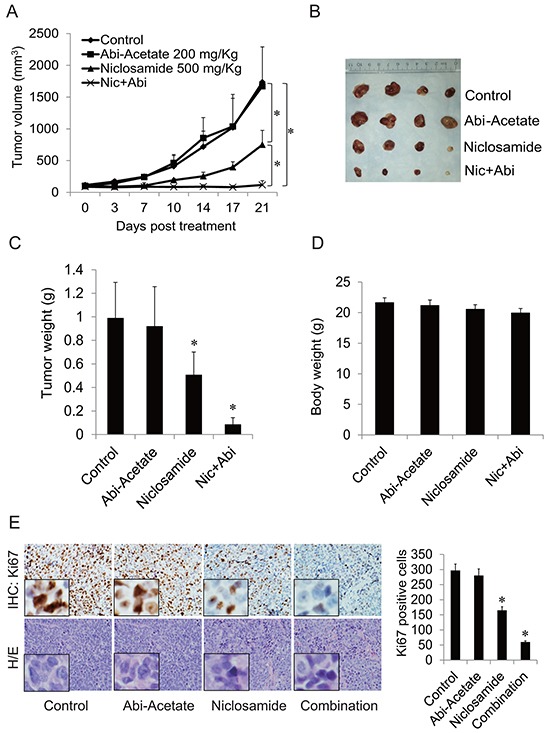
Niclosamide enhanced abiraterone treatment *in vivo* **A.** Mice bearing CWR22Rv1 xenografts were treated with vehicle control, abiraterone acetate (200mg/Kg orally), niclosamide (500 mg/Kg orally) or their combination for 3 weeks, tumor volumes were measured twice every week and the tumors were collected. **B.** Pictures of tumors from each group were taken after 3 weeks treatment. **C–D.** Each group tumor weight and body weight were measured and averaged. **E.** Ki67 was analyzed in tumor tissues by IHC staining and quantified as described in methods. *P<0.05 Abi-Acetate: Abiraterone Acetate.

To test the combination effects *in vivo*, a CWR22Rv1 xenograft model was used. Mice were treated with abiraterone acetate, niclosamide or their combination through oral administration. As shown in Figure 5A–5D, CWR22Rv1 tumors were resistant to abiraterone and treatment with niclosamide alone inhibited tumor growth. However, combination of niclosamide with abiraterone synergistically inhibited tumor size and tumor weight without decreasing mouse body weights. The cell proliferation rate of tumor samples was analyzed by IHC for Ki67 expression. Niclosamide inhibited Ki67 expression while combination treatment with abiraterone further decreased Ki67 expression (Figure 5E). These data demonstrate that niclosamide synergizes with abiraterone treatment in abiraterone resistant CWR22Rv1 tumor models through oral administration, and suggest that inhibiting AR-V7 with niclosamide is a potent treatment strategy for advanced prostate cancer.

## DISCUSSION

Since abiraterone and enzalutamide were approved by the FDA, the great potential to extend the lives and improve the quality of advanced prostate cancer patients is on horizon. Unfortunately, resistance to abiraterone and enzalutamide occurs frequently and there is still no definitive cure for metastatic CRPC patients. Considerable evidence from both clinical and experimental studies suggests that androgen receptor variants, particularly AR-V7, play vital roles in the development of resistance to enzalutamide as well as abiraterone therapy [14, 16, 23–26]. In the present study, we demonstrated that AR-V7 confers resistance to abiraterone and that niclosamide, a previously identified potent inhibitor of AR-V7, re-sensitizes resistant cells to abiraterone treatment. Furthermore, niclosamide synergizes with abiraterone treatment both *in vitro* and *in vivo*.

Both abiraterone and enzalutamide are FDA approved drugs. When prostate cancer becomes resistant to one drug, the subsequent response rate to the other drug is 20% or less [8, 30, 31], suggesting cross-resistance exists between these two drugs. However, the underlying mechanisms are incompletely understood. The role of AR splice variants in CRPC has been functionally characterized in recent years due to their prevalence in advanced prostate cancer tissues [21, 32, 33]. Truncated AR variants including AR-V7 have been suggested to drive castration resistant growth and AR target genes expression leading to resistance to enzalutamide [23, 24]. In the present study, we showed that cells which are resistant to enzalutamide are also resistant to abiraterone, and that AR-V7 overexpression confers resistance to abiraterone. Chronic treatment of C4-2B cells with abiraterone significantly induced AR-V7 protein expression; while overexpression of AR-V7 in C4-2 cells drives these cells become resistant to abiraterone. Knock down of AR-V7 expression by siRNA in CWR22Rv1, C4-2B MDVR and C4-2B AbiR cells significantly restored their sensitivity to abiraterone treatment, suggesting that AR-V7 plays an important role in the development of abiraterone resistance in addition to enzalutamide resistance.

Abiraterone inhibits CYP17A1 and reduces circulating androgen levels that activate the full length AR. A recent report also showed that abiraterone could be converted to D4A, a more active form which antagonizes full length AR directly [3]. While AR-V7 overexpression confers resistance to current anti-androgen therapies, AR-V7 is not suppressed by abiraterone. Therefore, there is an urgent need to develop novel agents that inhibit AR-V7 to overcome drug resistance. Our previous studies identified niclosamide as a potent inhibitor of AR-V7, and found that it significantly enhances enzalutamide treatment both *in vitro* and *in vivo* [27]. Niclosamide is an FDA approved drug for the treatment of human tapeworm infection and has rich repository of pharmacokinetic data [34–36]. Niclosamide has been proven to be safe and has very low toxicity in patients [37–41], and therapeutic blood concentrations can be achieved through oral administration using FDA approved administration dose. In the present study, we showed that niclosamide enhanced abiraterone treatment through oral administration in prostate cancer xenograft model, suggesting the combination of niclosamide and abiraterone warrants clinical investigation to treat advanced prostate cancer patients.

Taken together, we found that AR-V7 is involved in the cross resistance of enzalutamide and abiraterone. Targeting AR-V7 by siRNA or inhibition of protein expression by niclosamide can significantly enhance abiraterone treatment *in vitro* and *in vivo*. These data support the combination of abiraterone and niclosamide as a potential treatment for advanced prostate cancer.

## MATERIALS AND METHODS

### Reagents and cell culture

LNCaP, C4-2, C4-2B and CWR22Rv1 cells were maintained in RPMI 1640 supplemented with 10% fetal bovine serum (FBS), 100 units/ml penicillin and 0.1 mg/ml streptomycin. C4-2-neo and C4-2 AR-V7 cells were generated by stable transfection of C4-2 cells with either empty vector pcDNA3.1 or pcDNA3.1 encoding AR-V7 and were maintained in RPMI1640 medium containing 300 μg/mL G418. C4-2B MDVR (C4-2B enzalutamide resistant) cells were described previously [29]. C4-2B MDVR cells were maintained in 20 μM enzalutamide containing medium. C4-2B AbiR (C4-2B abiraterone resistant) cells were cultured in 5-20 μM abiraterone acetate over 12 months and maintained in 10 μM abiraterone acetate containing medium. All cells were maintained at 37°C in a humidified incubator with 5% carbon dioxide.

### Plasmids transfection and luciferase assay

For small interfering RNA (siRNA) transfection, cells were seeded at a density of 1×10^5^ cells per well in 12-well plates or 3×10^5^ cells per well in 6-well plates and transfected with siRNA (Dharmacon) targeting the AR-V7 sequence (GUAGUUGUAAGUAUCAUGA) [42] or a control siRNA targeting the luciferase (Luc) gene, non-targeting siRNA control (CTTACGCTGAGTACTTCGA), using Lipofectamine 2000 (Invitrogen).

### Western blot analysis

Cellular protein extracts were resolved on SDS–PAGE and proteins were transferred to nitrocellulose membranes. After blocking for 1 hour at room temperature in 5% milk in PBS/0.1% Tween-20, membranes were incubated overnight at 4°C with the indicated primary antibodies [AR441, (SC-7305, Santa Cruz Biotechnology, Santa Cruz, CA); AR-V7 (AG10008, Precision antibody); Tubulin (T5168, Sigma-Aldrich, St. Louis, MO)]. Tubulin was used as loading control. Following secondary antibody incubation, immunoreactive proteins were visualized with an enhanced chemiluminescence detection system (Millipore, Billerica, MA) [43].

### Cell growth assay

C4-2B parental or C4-2B MDVR cells were seeded on 12-well plates at a density of 1×10^5^ cells/well in RPMI 1640 media containing 10% FBS and treated with varying concentrations of abiraterone or enzalutamide for 48 hours; C4-2 neo, C4-2 AR-V7, LNCaP, C4-2B or CWR22Rv1 cells were treated with varying concentrations of abiraterone for 48 hours; CWR22Rv1, C4-2B MDVR or C4-2B AbiR cells were transiently transfected with siRNA targeting AR-V7 or a control siRNA, followed by treatment with abiraterone for 3 days. CWR22Rv1 cells, C4-2B MDVR or C4-2B AbiR cells were seeded on 12-well plates at a density of 0.5×10^5^ cells/well in RPMI 1640 media containing 10% FBS and co-treated with niclosamide and abiraterone in media containing FBS for 2 days. Total cell numbers were counted and the cell survival rate (%) was calculated. Cell survival rate (%) = (Treatment group cell number / Control group cell number) ×100%.

### Clonogenic assay

C4-2B parental or C4-2B MDVR cells were treated with DMSO, 5 μM, 10 μM or 20 μM abiraterone, or 10 μM or 20 μM enzalutamide in media containing 10% complete FBS. CWR22Rv1 cells, C4-2B MDVR or C4-2B AbiR cells were treated with niclosamide with or without abiraterone. Cells were plated at equal densities (2000 cells/dish) in 100 mm dishes for 14 days; the medium was changed every 7 days. The colonies were rinsed with PBS before staining with 0.5% crystal violet/4% formaldehyde for 30 min and the numbers of colonies were counted as described previously [44].

### *In vivo* tumorigenesis assay

CWR22Rv1 cells (4 million) were mixed with matrigel (1:1) and injected subcutaneously into the flanks of 6-7 week male SCID mice. Tumor-bearing mice (tumor volume around 50-100 mm^3^) were randomized into four groups (5 mice each group) and treated as follows: (1) vehicle control (0.5% weight/volume (w/v) Methocel A4M through oral), (2) Abiraterone acetate (200 mg/kg, through oral), (3) Niclosamide (500 mg/kg, through oral), (4) Abiraterone acetate (200 mg/kg, through oral) + Niclosamide (500 mg/kg, through oral). Tumors were measured using calipers twice a week and tumor volumes were calculated using length × width2/2. Tumor tissues were harvested after 3 weeks of treatment.

### Immunohistochemistry

Tumors were fixed by formalin and paraffin embedded. Tissue sections were dewaxed, rehydrated, and blocked for endogenous peroxidase activity. Antigen retrieval was performed in sodium citrate buffer (0.01 mol/L, pH 6.0) in a microwave oven at 1,000 W for 3 min and then at 100 W for 20 min. Nonspecific antibody binding was blocked by incubating slides in 10% fetal bovine serum in PBS for 30 min at room temperature. Slides were then incubated with anti-Ki-67 (at 1:500; NeoMarker) at room temperature for 30 min. Slides were then washed and incubated with biotin-conjugated secondary antibodies for 30 min, followed by incubation with avidin DH-biotinylated horseradish peroxidase complex for 30 min (Vectastain ABC Elite Kit, Vector Laboratories). The sections were developed with the diaminobenzidine substrate kit (Vector Laboratories) and counterstained with hematoxylin. Nuclear staining of cells was scored and counted in 5 different areas of the tissue section. Images were taken with an Olympus BX51 microscope equipped with DP72 camera.

### Statistical analysis

All data are presented as means ± standard deviation of the mean (SD). Statistical analyses were performed with Microsoft Excel analysis tools. Differences between individual groups were analyzed by one-way analysis of variance (ANOVA) followed by the Scheffé procedure for comparison of means. *P* < 0.05 was considered statistically significant.

## References

[1] Attard G, Reid AH, Olmos D, de Bono JS (2009). Antitumor activity with CYP17 blockade indicates that castration-resistant prostate cancer frequently remains hormone driven. Cancer research.

[2] Attard G, Reid AH, A'Hern R, Parker C, Oommen NB, Folkerd E, Messiou C, Molife LR, Maier G, Thompson E, Olmos D, Sinha R, Lee G (2009). Selective inhibition of CYP17 with abiraterone acetate is highly active in the treatment of castration-resistant prostate cancer. Journal of clinical oncology.

[3] Li Z, Bishop AC, Alyamani M, Garcia JA, Dreicer R, Bunch D, Liu J, Upadhyay SK, Auchus RJ, Sharifi N (2015). Conversion of abiraterone to D4A drives anti-tumour activity in prostate cancer. Nature.

[4] de Bono JS, Logothetis CJ, Molina A, Fizazi K, North S, Chu L, Chi KN, Jones RJ, Goodman OB, Saad F, Staffurth JN, Mainwaring P, Harland S (2011). Abiraterone and increased survival in metastatic prostate cancer. The New England journal of medicine.

[5] Ryan CJ, Smith MR, de Bono JS, Molina A, Logothetis CJ, de Souza P, Fizazi K, Mainwaring P, Piulats JM, Ng S, Carles J, Mulders PF, Basch E (2013). Abiraterone in metastatic prostate cancer without previous chemotherapy. The New England journal of medicine.

[6] Ryan CJ, Smith MR, Fizazi K, Saad F, Mulders PF, Sternberg CN, Miller K, Logothetis CJ, Shore ND, Small EJ, Carles J, Flaig TW, Taplin ME (2015). Abiraterone acetate plus prednisone versus placebo plus prednisone in chemotherapy-naive men with metastatic castration-resistant prostate cancer (COU-AA-302): final overall survival analysis of a randomised, double-blind, placebo-controlled phase 3 study. The Lancet Oncology.

[7] Scher HI, Fizazi K, Saad F, Taplin ME, Sternberg CN, Miller K, de Wit R, Mulders P, Chi KN, Shore ND, Armstrong AJ, Flaig TW, Flechon A (2012). Increased survival with enzalutamide in prostate cancer after chemotherapy. The New England journal of medicine.

[8] Bianchini D, Lorente D, Rodriguez-Vida A, Omlin A, Pezaro C, Ferraldeschi R, Zivi A, Attard G, Chowdhury S, de Bono JS (2014). Antitumour activity of enzalutamide (MDV3100) in patients with metastatic castration-resistant prostate cancer (CRPC) pre-treated with docetaxel and abiraterone. European journal of cancer.

[9] Loriot Y, Bianchini D, Ileana E, Sandhu S, Patrikidou A, Pezaro C, Albiges L, Attard G, Fizazi K, De Bono JS, Massard C (2013). Antitumour activity of abiraterone acetate against metastatic castration-resistant prostate cancer progressing after docetaxel and enzalutamide (MDV3100). Annals of oncology.

[10] Noonan KL, North S, Bitting RL, Armstrong AJ, Ellard SL, Chi KN (2013). Clinical activity of abiraterone acetate in patients with metastatic castration-resistant prostate cancer progressing after enzalutamide. Annals of oncology.

[11] Schrader AJ, Boegemann M, Ohlmann CH, Schnoeller TJ, Krabbe LM, Hajili T, Jentzmik F, Stoeckle M, Schrader M, Herrmann E, Cronauer MV (2014). Enzalutamide in castration-resistant prostate cancer patients progressing after docetaxel and abiraterone. European urology.

[12] Thomsen FB, Roder MA, Rathenborg P, Brasso K, Borre M, Iversen P (2013). Enzalutamide treatment in patients with metastatic castration-resistant prostate cancer progressing after chemotherapy and abiraterone acetate. Scandinavian journal of urology.

[13] van Soest RJ, van Royen ME, de Morree ES, Moll JM, Teubel W, Wiemer EA, Mathijssen RH, de Wit R, van Weerden WM (2013). Cross-resistance between taxanes and new hormonal agents abiraterone and enzalutamide may affect drug sequence choices in metastatic castration-resistant prostate cancer. European journal of cancer.

[14] Mostaghel EA, Marck BT, Plymate SR, Vessella RL, Balk S, Matsumoto AM, Nelson PS, Montgomery RB (2011). Resistance to CYP17A1 inhibition with abiraterone in castration-resistant prostate cancer: induction of steroidogenesis and androgen receptor splice variants. Clinical cancer research.

[15] Cai C, Chen S, Ng P, Bubley GJ, Nelson PS, Mostaghel EA, Marck B, Matsumoto AM, Simon NI, Wang H, Chen S, Balk SP (2011). Intratumoral de novo steroid synthesis activates androgen receptor in castration-resistant prostate cancer and is upregulated by treatment with CYP17A1 inhibitors. Cancer research.

[16] Yu Z, Chen S, Sowalsky AG, Voznesensky OS, Mostaghel EA, Nelson PS, Cai C, Balk SP (2014). Rapid induction of androgen receptor splice variants by androgen deprivation in prostate cancer. Clinical cancer research.

[17] Azad AA, Volik SV, Wyatt AW, Haegert A, Le Bihan S, Bell RH, Anderson SA, McConeghy B, Shukin R, Bazov J, Youngren J, Paris P, Thomas G (2015). Androgen Receptor Gene Aberrations in Circulating Cell-Free DNA: Biomarkers of Therapeutic Resistance in Castration-Resistant Prostate Cancer. Clinical cancer research.

[18] Chen EJ, Sowalsky AG, Gao S, Cai C, Voznesensky O, Schaefer R, Loda M, True LD, Ye H, Troncoso P, Lis RL, Kantoff PW, Montgomery RB (2015). Abiraterone treatment in castration-resistant prostate cancer selects for progesterone responsive mutant androgen receptors. Clinical cancer research.

[19] Li Y, Alsagabi M, Fan D, Bova GS, Tewfik AH, Dehm SM (2011). Intragenic rearrangement and altered RNA splicing of the androgen receptor in a cell-based model of prostate cancer progression. Cancer research.

[20] Dehm SM, Tindall DJ (2011). Alternatively spliced androgen receptor variants. Endocrine-related cancer.

[21] Hu R, Dunn TA, Wei S, Isharwal S, Veltri RW, Humphreys E, Han M, Partin AW, Vessella RL, Isaacs WB, Bova GS, Luo J (2009). Ligand-independent androgen receptor variants derived from splicing of cryptic exons signify hormone-refractory prostate cancer. Cancer research.

[22] Zhang Z, Zhou N, Huang J, Ho TT, Zhu Z, Qiu Z, Zhou X, Bai C, Wu F, Xu M, Mo YY (2016). Regulation of androgen receptor splice variant AR3 by PCGEM1. Oncotarget.

[23] Nadiminty N, Tummala R, Liu C, Yang J, Lou W, Evans CP, Gao AC (2013). NF-kappaB2/p52 induces resistance to enzalutamide in prostate cancer: role of androgen receptor and its variants. Molecular cancer therapeutics.

[24] Li Y, Chan SC, Brand LJ, Hwang TH, Silverstein KA, Dehm SM (2013). Androgen receptor splice variants mediate enzalutamide resistance in castration-resistant prostate cancer cell lines. Cancer research.

[25] Steinestel J, Luedeke M, Arndt A, Schnoeller TJ, Lennerz JK, Wurm C, Maier C, Cronauer MV, Steinestel K, Schrader AJ (2015). Detecting predictive androgen receptor modifications in circulating prostate cancer cells. Oncotarget.

[26] Antonarakis ES, Lu C, Wang H, Luber B, Nakazawa M, Roeser JC, Chen Y, Mohammad TA, Chen Y, Fedor HL, Lotan TL, Zheng Q, De Marzo AM (2014). AR-V7 and resistance to enzalutamide and abiraterone in prostate cancer. The New England journal of medicine.

[27] Liu C, Lou W, Zhu Y, Nadiminty N, Schwartz CT, Evans CP, Gao AC (2014). Niclosamide Inhibits Androgen Receptor Variants Expression and Overcomes Enzalutamide Resistance in Castration-Resistant Prostate Cancer. Clinical Cancer Research.

[28] Liu C, Lou W, Armstrong C, Zhu Y, Evans CP, Gao AC (2015). Niclosamide suppresses cell migration and invasion in enzalutamide resistant prostate cancer cells via Stat3-AR axis inhibition. The Prostate.

[29] Liu C, Lou W, Zhu Y, Yang JC, Nadiminty N, Gaikwad NW, Evans CP, Gao AC (2015). Intracrine Androgens and AKR1C3 Activation Confer Resistance to Enzalutamide in Prostate Cancer. Cancer research.

[30] Azad AA, Eigl BJ, Murray RN, Kollmannsberger C, Chi KN (2015). Efficacy of enzalutamide following abiraterone acetate in chemotherapy-naive metastatic castration-resistant prostate cancer patients. European urology.

[31] Scher HI, Fizazi K, Saad F, Taplin M-E, Sternberg CN, Miller K, de Wit R, Mulders P, Chi KN, Shore ND, Armstrong AJ, Flaig TW, Fléchon A (2012). Increased Survival with Enzalutamide in Prostate Cancer after Chemotherapy. New England Journal of Medicine.

[32] Guo Z, Yang X, Sun F, Jiang R, Linn DE, Chen H, Chen H, Kong X, Melamed J, Tepper CG, Kung HJ, Brodie AM, Edwards J (2009). A novel androgen receptor splice variant is up-regulated during prostate cancer progression and promotes androgen depletion-resistant growth. Cancer research.

[33] Hu R, Lu C, Mostaghel EA, Yegnasubramanian S, Gurel M, Tannahill C, Edwards J, Isaacs WB, Nelson PS, Bluemn E, Plymate SR, Luo J (2012). Distinct transcriptional programs mediated by the ligand-dependent full-length androgen receptor and its splice variants in castration-resistant prostate cancer. Cancer research.

[34] Hayes WJ, Laws E.R (1991). Handbook of Pesticide Toxicology Classes of Pesticides.

[35] Andrews P, Thyssen J, Lorke D (1982). The biology and toxicology of molluscicides, Bayluscide. Pharmacology & therapeutics.

[36] Li YH, Li PK, Roberts MJ, Arend RC, Samant RS, Buchsbaum DJ (2014). Multi-targeted therapy of cancer by niclosamide: A new application for an old drug. Cancer Lett.

[37] Anon (1988). Drugs for parasitic infections. The Medical letter on drugs and therapeutics.

[38] Ronald NC, Wagner JE (1975). Treatment of Hymenolepis nana in hamsters with Yomesan (niclosamide). Laboratory animal science.

[39] Most H, Yoeli M, Hammond J, Scheinesson GP (1971). Yomesan (niclosamide) therapy of Hymenolepis nana infections. The American journal of tropical medicine and hygiene.

[40] Jones WE (1979). Niclosamide as a treatment for Hymenolepis diminuta and Dipylidium caninum infection in man. The American journal of tropical medicine and hygiene.

[41] Al-Hadiya BM (2005). Niclosamide: comprehensive profile. Profiles of drug substances, excipients, and related methodology.

[42] Hu R, Lu C, Mostaghel EA, Yegnasubramanian S, Gurel M, Tannahill C, Edwards J, Isaacs WB, Nelson PS, Bluemn E, Plymate SR, Luo J (2013). Distinct transcriptional programs mediated by the ligand-dependent full-length androgen receptor and its splice variants in castration-resistant prostate cancer. Cancer research.

[43] Liu C, Zhu Y, Lou W, Cui Y, Evans CP, Gao AC (2014). Inhibition of constitutively active Stat3 reverses enzalutamide resistance in LNCaP derivative prostate cancer cells. The Prostate.

[44] Liu C, Zhu Y, Lou W, Nadiminty N, Chen X, Zhou Q, Shi XB, deVere White RW, Gao AC (2013). Functional p53 determines docetaxel sensitivity in prostate cancer cells. The Prostate.

