# Correction: Niclosamide enhances abiraterone treatment via inhibition of androgen receptor variants in castration resistant prostate cancer

**DOI:** 10.18632/oncotarget.28656

**Published:** 2024-10-01

**Authors:** Chengfei Liu, Cameron Armstrong, Yezi Zhu, Wei Lou, Allen C. Gao

**Affiliations:** ^1^Department of Urology, University of California Davis, CA, USA; ^2^Graduate Program in Pharmacology and Toxicology, University of California Davis, CA, USA; ^3^UC Davis Comprehensive Cancer Center, University of California Davis, CA, USA

**This article has been corrected:** In Figure 5E, row H/E, the image in the ‘Abi-Acetate’ panel is an accidental duplicate of the image found in the ‘Control’ panel. The corrected Figure 5E, obtained using the original data, is shown below. The authors declare that these corrections do not change the results or conclusions of this paper.

Original article: Oncotarget. 2016; 7:32210–32220. 32210-32220. https://doi.org/10.18632/oncotarget.8493

**Figure 5:**
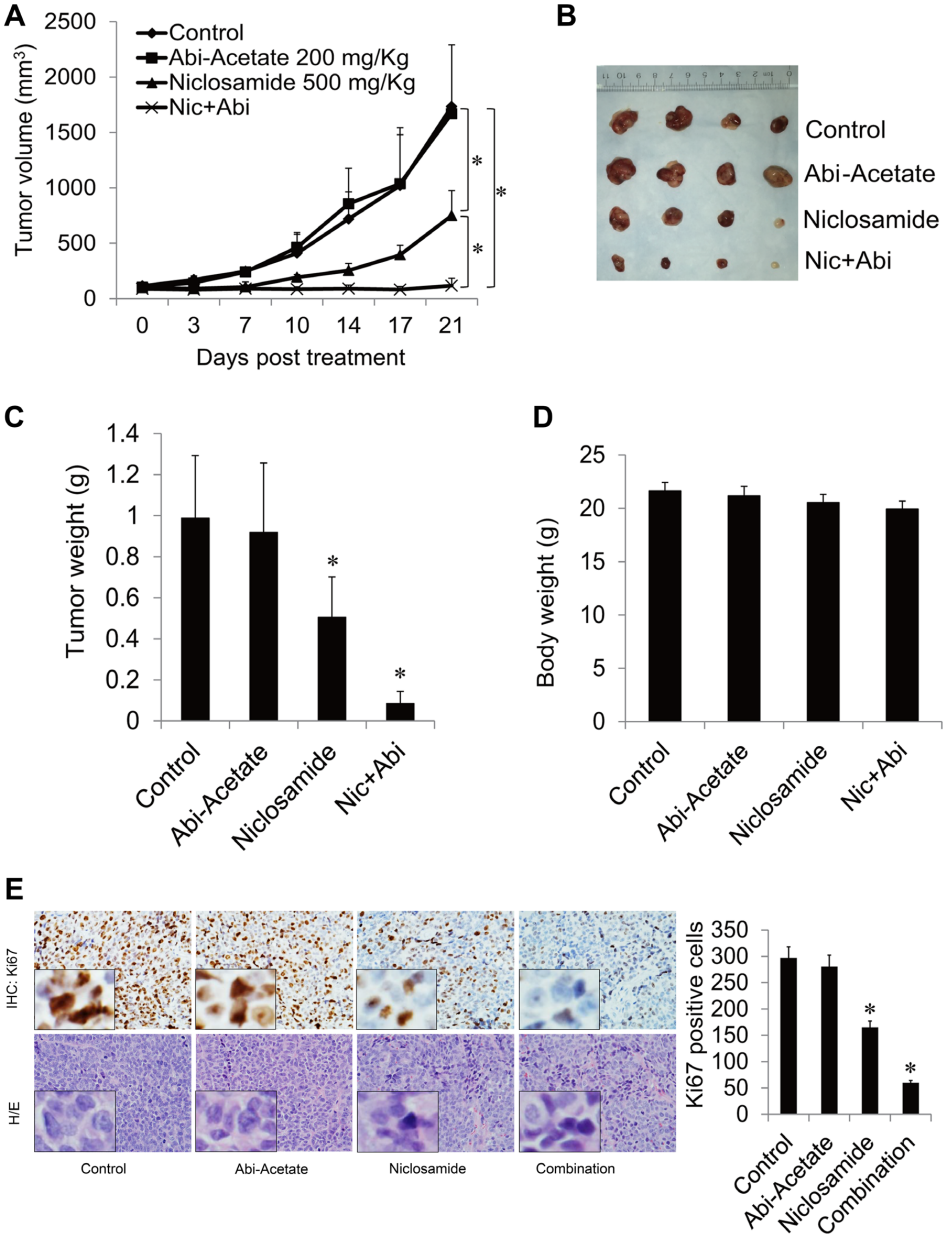
Niclosamide enhanced abiraterone treatment *in vivo*. (**A**) Mice bearing CWR22Rv1 xenografts were treated with vehicle control, abiraterone acetate (200 mg/Kg orally), niclosamide (500 mg/Kg orally) or their combination for 3 weeks, tumor volumes were measured twice every week and the tumors were collected. (**B**) Pictures of tumors from each group were taken after 3 weeks treatment. (**C**, **D**) Each group tumor weight and body weight were measured and averaged. (**E**) Ki67 was analyzed in tumor tissues by IHC staining and quantified as described in methods. ^*^ *P* < 0.05. Abbreviation: Abi-Acetate: Abiraterone Acetate.

